# Expert Consensus on the Treatment of Hypertension with Chinese Patent Medicines

**DOI:** 10.1155/2013/510146

**Published:** 2013-04-03

**Authors:** Li Ying Wang, Kam Wa Chan, Ya Yuwen, Nan Nan Shi, Xue Jie Han, Aiping Lu

**Affiliations:** ^1^Institute of Basic Research in Clinical Medicine, China Academy of Chinese Medical Sciences, Beijing 100700, China; ^2^Tung Wah Group of Hospitals, Hong Kong Special Administrative Region, Hong Kong; ^3^School of Chinese Medicine, Hong Kong Baptist University, Hong Kong Special Administrative Region, Hong Kong

## Abstract

*Objectives*. This study was aimed to determine the therapeutic principle and identify Chinese Patent Medicine (CPM) with corresponding indications for hypertension treatment. *Methods*. Three rounds of Delphi survey were mailed among 40 cardiovascular integrative medicine specialists. Items with agreement of more than 80% respondents were included in the consensus. *Results*. According to majority of the panelists, CPM is suitable for most hypertensive patients and should be used according to traditional Chinese medicine pattern classification. CPM could be used alone for grade 1 hypertension and could be used in combination with Western biomedicine (WM) for both grade 2 and grade 3 hypertension. It is recommended that less than two CPMs are used simultaneously. For the treatment of grade 2 and 3 hypertension, CPM and WM should be taken separately. Recommended CPMs included Tianma Gouteng granule, Qiju Dihuang capsule, Jinkui Shenqi pill, Yinxingye tablet, Niuhuang Jiangya pill and Banxia Tianma pill. The indications of 4 CPMs were specified with symptoms related to TCM pattern classification by the experts. *Conclusions*. An expert consensus on CMP application was formed for the treatment of hypertension in the form of integrative medicine. A flow of IM hypertension management was proposed based on the results of the survey.

## 1. Introduction

Hypertension is an alarming global health issue with increasing longevity and prevalent contributing factors, such as obesity, physical inactivity, and an unhealthy diet [1–3]. It is of highest morbidity rate of chronic diseases in China [4]. The current prevalence in many developing countries is reaching that of developed countries [5, 6]. 

TCM has been used widely in treating hypertension in China [7], and recently more Chinese patent medicines (CPMs) were developed for convenient application and better quality control [8–13]. Currently, there are 86 CPMs available for the treatment of hypertension as listed in the Chinese pharmacopoeia (2010 edition) [14]. These CPMs were used not only by TCM doctors but also by WM doctors. Since most of the WM doctors have inadequate training in TCM, the misuse of CPMs is a practical concern. 

Different CPMs should be used to treat diseases with different TCM patterns according to TCM pattern differentiation theory. In China, there are no mandatory guidelines for the application of CPMs as a treatment for hypertension. We aimed to develop a guideline for best practice of using CPM to treat hypertension through a Delphi survey among a cohort of cardiovascular experts in IM. 

Delphi surveys are commonly used to reach agreement of clinical protocols [15]. A Delphi survey involves a group of experts with equal opportunity to contribute to decision making in an anonymous, iterative, regulated, and statistically assisted manner [16]. This process prevents negative effects of group interactions including dominance of vocal group members and imposes few geographical constraints [17]. To ensure easy understanding and application among doctors without TCM training, we presented the indications of CPM with common symptoms.

## 2. Methods

### 2.1. Participating Experts

A clinical expert consensus management group was formed. 40 IM cardiovascular experts were selected with three criteria: (1) with more than ten years clinical experience in the treatment of hypertension with CPMs; (2) have published at least 4 peer reviewed papers related to the treatment of hypertension with IM; (3) recommended by the Cardiovascular Disease Division of China Association of Chinese Medicine. 

### 2.2. Questionnaire

For the first round of survey, a questionnaire focused on the general therapeutic principle was deliberated among and set by the management group. A list of top 20 commonly used CPMs was generated from a systematic literature search in PubMed and Chinese CNKI. All these included 33 items covering the following areas: the general principle of using CPMs, the commonly used CPMs and the corresponding indications.

Experts were invited to add any comments that they perceived to be necessary and the CPMs that they used frequently in their clinical practice. 

### 2.3. Delphi Process

Three rounds of consultation were carried out in this study. The questionnaire was mailed to the panelists. A month after the deadline of response, a reminder was sent to experts who did not respond.

In the first round, the experts were invited to finish the questionnaire and add the general principles of CPMs application and CPMs they commonly used in treatment of hypertension. The results were collected and submitted to the management group. 

After analysis and discussion, a revised questionnaire for the second round consultation was generated and subsequently disseminated to the experts who responded in the first round. Items were accepted as consensus directly when 80% of respondents reached agreement. Items receiving less than 20% support were removed. Items receiving between 20% and 80% were recirculated to the next round consultation. A list of top 20 commonly used CPMs together with the CPMs suggested by the experts were included. The third round of questionnaire was composed of the same questions which did not achieve consensus in previous consultation. All respondents had access of second round scores of the whole group to provide information for consensus items from the second round and achieve the final consensus. As it is well understood that the application of CPMs varies among different CM doctors and different pattern of disease, we included CPMs with consensus over 70% so as to provide more options on treatment. Corresponding confidence intervals were shown as reference.

## 3. Results

### 3.1. Participants

The questionnaire was mailed to the 40 experts satisfying the inclusion criteria. All of them completed the first round of consultation, 37 experts completed the second round, and 33 experts finished the third round. The panelists aged from 37 to 70 and were all ranked of professor or associate professor.  Table 1 shows the demographics of the panelists.

### 3.2. Items Endorsed in the Consensus

**Figure 1:**
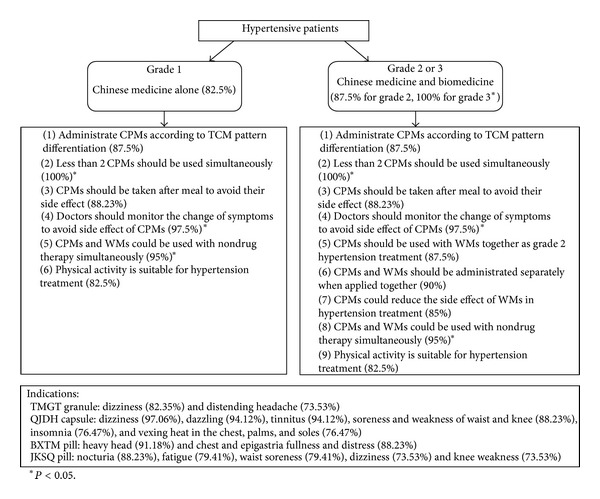
Proposed flow of hypertension management in the integrative medicine perspective.

After three rounds of consultation, the items with consensus included 12 general therapeutic principles of CPMs application, six CPMs with indications and precautions.  Figure 1 shows the management flow of hypertension with CPMs based on the results in the survey.

In the first round of consultation, 21 items on general therapeutic principle and 20 CPMs for the treatment of hypertension were collected from literature review and constituted the questionnaire for the first round of consultation. The questionnaires were mailed to the panelists. Four items which reached consensus from more than 80% experts were accepted directly (Table 2), including “CPMs and WMs should be used according to hypertension grades in WM diagnosis,” “CPMs could reduce the side effect of western medicines in hypertension treatment,” “CPMs and WMs could be used in combination with other types of TCM non-drug therapy,” and “Physical activity is suitable for hypertension treatment”.

In the second round of consultation, 7 items on general therapeutic principle reached consensus from more than 80% experts (Table 2), including “CPMs could be used alone in grade 1 hypertension treatment,” “CPMs should be used according to TCM pattern classification,” “Less than two kinds of CPMs were recommended to be used simultaneously,” “Doctors should monitor the symptom changes to avoid side effect of CPMs,” “CPMs should be used with WMs together in grade 2 and grade 3 hypertension treatment,” “CPMs and WMs should be taken separately when used together” (Table 2). 

In the third round of consultation, 1 more item of the general therapeutic principle for CPM application was selected by more than 80% experts, and 6 kinds of CPMs were selected by 70% or more experts (Table 2). Selected CPMs included Tianma Gouteng (TMGT) granule (90.00%), Qiju Dihuang (QJDH) capsule (87.50%), Jinkui Shenqi (JKSQ) pill (80.00%), Yinxingye (YXY) tablet (80.00%), Niuhuang Jiangya (NHJY) pill (76.47%), and Banxia Tianma (BXTM) Pill (70.59%). Detailed information about the 6 selected CPMs were shown in Table 3. In addition, the specific indication based on TCM symptoms of 4 selected CPMs was identified by more than 70% experts and shown in Table 4.

## 4. Discussion

We aimed to reach an expert consensus on CPMs treatment for hypertension for WM doctors who intend to use CPMs in hypertension management. We used the Delphi method which enabled us to utilize both the research and clinical experience of the experts who participated with no geographical limitations. All panelists of the consultation were IM cardiologist. They were all recommended by the Cardiovascular Disease Division of China Association of Chinese Medicine. Compared with the TCM doctors who use herbal decoctions more frequently than CPMs, IM doctors treat hypertension with both CPMs and WMs simultaneously more often. We selected IM doctors as they had both WM and CM education background as well as clinical experience on the use of both CPMs and WMs. We believe that they are more capable to explain with TCM terms and have richer experience on clinical use of related CPMs and WMs. 

The consensus included twelve general therapeutic principles of CPMs and six CPMs with corresponding indications. Majority agreed that CPMs could be used for most patients with hypertension and the application varies among different stages of hypertension. CPM could be used alone in grade 1 hypertension and should be used along with WM for grade 2 and grade 3 hypertension. The results are coherent with the clinical practice in China currently. Previous studies showed that CPMs have similar effect in treating grade 1 hypertension as WMs [18]. Furthermore, the clinical effectiveness is enhanced with the concurrent use of WMs in grade 2 and grade 3 hypertension management [19, 20]. Therefore, CPMs should be used with WMs together when treating grade 2 and grade 3 hypertensive patients. 

CPMs are developed based on TCM theory as a result, they should be used according to TCM pattern classification in the treatment of hypertension. Hypertensive patients usually presented with headache and dizziness, and sometimes tinnitus, soreness and weakness of waist and knee, and fear of cold and cold limbs. All these could be classified into different patterns. Studies have shown the correlation between hypertension and different TCM patterns [21, 22] with typical symptoms. In this study, we listed a wide range of symptoms for pattern classification in the consultation in order to yield symptom-based indications of recommended CPMs. 

Hypertension is associated with multiple cardiovascular risk factors, and the concurrent use of multiple CPMs for hypertension treatment is a common practice. According to most of the IM or TCM experts, simultaneous application of CPMs may have an additive effect. However, in recent years, there have been an increasing number of reports on the side effect of concurrent use of CPMs [23]. To avoid the side effects, experts recommended that less than two CPMs should be used simultaneously. In addition, CPMs should be taken after meal, and the practitioners should monitor the change of symptoms to avoid the side effects. 

The concurrent use of TCM and WM has been popular in China which leads to a new medical model known as integrated traditional and western medicine or integrative medicine. With regard to hypertension treatment, this approach is being used clinically, involving CPMs and different categories of WMs such as diuretics, Beta-blockers, CCBs, and ACEIs [24]. It is shown in many studies that combined treatments are more effective in disease control and symptoms modification when compared with single treatment [25]. 

In this survey, 87.5% of experts agreed that CPMs and WMs should be administered separately in the treatment of grade 2 and grade 3 hypertension. It is sensible to take CPMs and WMs apart in clinical practice, as CPMs are complex and there may be interaction between the medicines. Some studies showed that CPMs and Chinese herbal medicines (CHM) have the effect of reducing side effects of WMs in hypertension treatment [26], and 85% of the panelists agreed with that. 

A range of lifestyle modifications have been shown in clinical trials with the effect of lowering blood pressure and reducing the incidence of hypertension, similar to majority (82.5%) of the panelists' viewpoint. Physical activity is one of the most important among them [27, 28].

In this study, more than 70% of the experts selected six CPMs for hypertension treatment including TMGT, QJDH JKSQ, YXY, NHJY, and BXTM. TMGT, QJDH, YXY, NHJY and BXTM were recorded in the Chinese pharmacopoeia (2010 edition). TMGT granule, with highest consensus and the only CPM selected by more than 90% of panelists, is a recommended treatment in the clinical practice guideline for hypertension in TCM [9]. TMGT granule is composed of the herbs Gastrodiae Rhizoma (Tian Ma), Uncariae Ramulus Cum Uncis (Gou Teng), Haliotidis Concha (Shi Jue Ming), Gardeniae Fructus (Zhi Zi), Astragali Radix (Huang Qi), Achyranthis Bidentatae Radix (Niu Xi), Eucommiae Cortex (Yan Du Zhong), Leonuri Herba (Yi Mu Cao), Taxilli Herba (Sang Ji Sheng), Polygoni Multiflori Caulis (Shou Wu Teng), and Poria (Fu Ling). The indication of TMGT granule includes headache, dizziness, tinnitus, dazzle, tremor, and insomnia, which can be categorized as liver-yang hyperactivity according to TCM pattern differentiation [14]. It has been proven that TMGT granule is effective in lowering blood pressure and modifying symptoms when used alone [29] and that is even enhanced when used with WM concurrently [30]. Pharmacological researches demonstrated that TMGT granule can attenuate myocardial and aorta hypertrophy induced by renovascular hypertension and suppress the rise of tissue Ang II significantly [31]. 

QJDH capsule consists of eight herbal medicines including Lycii Fructus (Gou Qi Zi), Mume Flos (Ju Hua), Rehmanniae Radix Praeparata (Shu Di Huang), Corni Fructus (Shan Zhu Yu), Moutan Cortex (Mu Dan Pi), Dioscoreae Rhizoma (Shan Yao), Poria (Fu Ling), and Aliamria Rhizoma (Yan Ze Xie). The indication of QJDH capsule includes vertigo, tinnitus, photophobia, and epiphora with wind and blurred vision, which can be categorized as liver-kidney yin deficiency [14]. It is shown that QJDH capsule is effective in regulating immunologic function and improving clinical symptoms [32]. The effect of controlling blood pressure is enhanced when QJDH capsule was used with verapamil simultaneously [33]. 

NHJY pill is composed of fourteen herbal medicines including Saigae Tataricae Cornu (Ling Yang Jiao), Margaritifera Concha (Zhen Zhu Mu), Bubali Cornu (Shui Niu Jiao), Bovis Calculus (Ren Gong Niu Huang), Borneolum Syntheticum (Bing Pian), Paeoniae Radix Alba (Bai Shao), Salviae Miltiorrhizae Radix Et Rhizoma (Dang Shen), Astragali Radix (Huang Qi), Cassiae Semen (Jue Ming Zi), Chuanxiong Rhizoma (Chuan Xiong), Scutellariae Radix (Huang Qin Ti Qu Wu), Nardostachyos Radix Et Rhizoma (Gan Song), Menthae Haplocalycis Herba (Bo He), and Curcumae Radix (Yu Jin). The indication of NHJY pill includes light headedness, headache, insomnia, and dysphoria which can be categorized as heat in heart and liver and phlegm heat obstruct [14]. Clinical studies demonstrated that NHJY pill is suitable for grade 1 and grade 2 hypertension treatments [34]. It can relieve headache, irritability, anxiety and depress sympathetic nervous activity [35]. NHJY pill includes many metallic elements such as sodium, calcium, magnesium, potassium, iron, chromium, manganese, nickel, cadmium, copper, and zinc [36]. The side effect of NHJY pill includes minor gastric discomfort which can be relieved after symptomatic treatment [37].

YXY tablet was mainly composed of flavonol glycosides, an extract of Chinese herbal medicine ginkgo leaf. The indications of YXY tablet are cardialgia, stroke, and hemiplegia, which can be categorized in TCM pattern as obstruction of collaterals by blood stasis [14]. Clinical studies showed that YXY tablet has good effect on lowering blood pressure and improving the quality of life for hypertensive patient when used alone or combined with WMs [38, 39]. Pharmacological research showed that it can inhibit hypertension ventricle remodeling by downregulating the serum TGF-p1 [40]. The side effect of YXY tablet includes urticaria, which would disappear after cessation of medication [41].

BXTM pill consists of Pinelliae Rhizoma Praeparatum (Fa Ban Xia), Gastrodiae Rhizoma (Tian Ma), Astragali Radix Praeparata Cum Melle (Zhi Huang Qi),Ginseng Radix Et Rhizoma (Ren Shen), Atractylodis Rhizoma (Cang Zhu), Atractylodis Macrocephalae Rhizoma (Chao Bai Zhu), Poria (Fu Ling), Citri Reticulatae Pericarpium (Chen Pi), Aliamria Rhizoma (Ze Xie), Massa Medicata Fermentata (Liu Shen Qu), Hordei Fructus Germinatus (Chao Mai Ya), and Phellodendri Chinensis Cortex (Huang Bai). Indication includes dizziness, headache, and chest and epigastric fullness and distress which can be categorized as damp abundance due to splenic asthenia and stagnation of endogenous retention of phlegm [14]. 

JKSQ pill is listed in the national essential medicine list and consists of Aconiti Lateralis Rddix Praeparata (Fu Zi), Cinnamomi Ramulus (Gui Zhi), Achyranthis Bidentatae Radix (Niu Xi), Rehmanniae Radix (Di Huang), Corni Fructus (Shan Zhu Yu), Dioscoreae Rhizoma, Poria (Fu Ling), Aliamria Rhizoma (Ze Xie), Plantaginis Semen (Che Qian Zi), and Moutan Cortex (Mu Dan Pi). The indication includes edema, soreness and weakness of waist and knees, dysuria, and extreme chilliness which can be categorized as deficiency of the kidney [14]. Clinical studies showed JKSQ pill has a high effective rate (91.25%) in hypertension treatment [42]. It also demonstrates promising effect on decreasing microalbuminuria when administered with WMs [43]. Side effect of JKSQ pill includes skin eruption, nausea, and abdominal pain [44]. 

There are several limitations in this study. First, it is difficult to achieve perfect consensus on CPMs for the treatment of hypertension, as there were many different CPMs available with different TCM pattern based indications. Also, they were in lack of evidence from quality trials. To provide maximum information for WM doctors, we accepted items that agreed by more than 70% of panelists in this study. 

Secondly, we did not use TCM pattern as the primary form of indication for CPMs. The aim of this study is to help WM doctors to apply CPMs for the treatment of hypertension accurately; therefore, we used clinical symptoms instead of TCM pattern as the form of indication. In TCM, pattern differentiation is based primarily on symptoms and signs including tongue appearance and pulse feelings. It would be hard for WM doctors to understand the context if the indications were illustrated with TCM patterns, as all these were derived from specialized TCM concepts. Lastly, because of inadequate related studies, we did not have consensus on the indication and possible side effect of YXY tablet and NHJY pill when used as the treatment of hypertension. 

In conclusion, we formed a consensus on the application of CPMs in the treatment of hypertension for WM doctors through three rounds of consultations. We believe it would help WM doctors to use CPMs more accurately in clinical hypertension management. We presented the indications in the form of typical symptoms instead of classical pattern differentiation in common TCM consensus or clinical practice guidelines (CPGs) as to assist the WM doctors or other doctors without TCM background to understand and apply CPMs easily. In addition to general therapeutic principles and specific CPM with indications, we also consulted the side effect of different CPMs so as to enhance the safety in clinical practice. This expert consensus could serve as the foundation of CPGs for treatment of hypertension in the future when more evidence on the treatment is established.

## Figures and Tables

**Table 1:** Demographics of panelists.

General information	Number (percentage)^∗1^
Age (years old)	
<40	1 (2.5%)
40~50	11 (27.5%)
50~60	24 (60.0%)
>60	4 (10.0%)
Ranking	
Professor	31 (77.5%)
Associate professor	9 (22.5%)
Clinical experience (years)	
10~20	9 (22.5%)
20~30	18 (45.0%)
≥30	13 (32.5%)

^∗1^The total number of panelists at round 1 is 40.

**Table 2:** General principles for hypertension treatment achieving consensus.

No.	Consensus	Round for consensus	Percentage of consensus (95% confidence interval)
1	CPMs and WMs could be used with nondrug therapy simultaneously	1	95.00% (82.6% to 100%)*
2	CPMs could reduce the side effect of WMs in hypertension treatment	1	85.00% (72.6% to 97.4%)
3	CPMs and WMs should be used according to WM diagnostic system	1	82.50 (70.1% to 94.9%)
4	Physical activity is suitable for hypertension treatment	1	82.50 (70.1% to 94.9%)
5	Less than two CPMs should be used simultaneously	2	100.00 (87.1% to 100%)*
6	CPMs should be used with WMs together as grade 3 hypertension treatment	2	100.00 (87.1% to 100%)*
7	Doctors should monitor the change of symptoms to avoid side effect of CPMs	2	97.50 (84.6% to 100%)*
8	CPMs and WMs should be administrated separately when applied together	2	90.00 (77.1% to 100%)
9	CPMs should be used with WMs together as grade 2 hypertension treatment	2	87.50 (74.6% to 100%)
10	CPMs should be used according to TCM pattern differentiation theory	2	87.50 (74.6% to 100%)
11	CPMs could be used alone as grade 1 hypertension treatment	2	82.50 (69.6% to 95.4%)
12	CPMs should be taken after meal to avoid their side effect	3	88.23 (74.6% to 100%)

*P < 0.05.

**Table 3:** General information on the CPMs achieving consensus.

Name	Source of information	Composition	Specification	Recommended dosage
Tianma Gouteng (TMGT) granule	Chinese pharmacopoeia (2010 edition)	Gastrodiae Rhizoma (Tian Ma), Uncariae Ramulus Cum Uncis (Gou Teng), Haliotidis Concha (Shi Jue Ming), Gardeniae Fructus (Zhi Zi), Astragali Radix (Huang Qi), Achyranthis Bidentatae Radix (Niu Xi), Eucommiae Cortex (Yan Du Zhong), Leonuri Herba (Yi Mu Cao), Taxilli Herba (Sang Ji Sheng), Polygoni Multiflori Caulis (Shou Wu Teng), and Poria (Fu Ling)	10 g/bag	10 g/t; t.i.d.

Qiju Dihuang (QJDH) capsule	Chinese pharmacopoeia (2010 edition)	Lycii Fructus (Gou Qi Zi), Mume Flos (Ju Hua), Rehmanniae Radix Praeparata (Shu Di Huang), Corni Fructus (Shan Zhu Yu), Moutan Cortex (Mu Dan Pi), Dioscoreae Rhizoma (Shan Yao), Poria (Fu Ling), and Aliamria Rhizoma (Yan Ze Xie).	0.3 g/granule	5-6 granules/t; t.i.d.

Niuhuang Jiangya (NHJY) pill	Chinese pharmacopoeia (2010 edition)	Saigae Tataricae Cornu (Ling Yang Jiao), Margaritifera Concha (Zhen Zhu Mu), Bubali Cornu (Shui Niu Jiao), Bovis Calculus (Ren Gong Niu Huang), Borneolum Syntheticum (Bing Pian), Paeoniae Radix Alba (Bai Shao), Salviae Miltiorrhizae Radix Et Rhizoma (Dang Shen), Astragali Radix (Huang Qi), Cassiae Semen (Jue Ming Zi), Chuanxiong Rhizoma (Chuan Xiong), Scutellariae Radix (Huang Qin Ti Qu Wu), Nardostachyos Radix Et Rhizoma (Gan Song), Menthae Haplocalycis Herba (Bo He), and Curcumae Radix (Yu Jin)	20 pills/1.3 g 1 pill/g	1.3 g–2.6 g/t; q.d. 1.6 g–3.2 g/t; q.d.

Banxia Tianma (BXTM) pill	Chinese pharmacopoeia (2010 edition)	Pinelliae Rhizoma Praeparatum (Fa Ban Xia), Gastrodiae Rhizoma (Tian Ma), Astragali Radix Praeparata Cum Melle (Zhi Huang Qi), Ginseng Radix Et Rhizoma (Ren Shen), Atractylodis Rhizoma (Cang Zhu), Atractylodis Macrocephalae Rhizoma (Chao Bai Zhu), Poria (Fu Ling), Citri Reticulatae Pericarpium (Chen Pi), Aliamria Rhizoma (Ze Xie), Massa Medicata Fermentata (Liu Shen Qu), Hordei Fructus Germinatus (Chao Mai Ya), and Phellodendri Chinensis Cortex (Huang Bai)	100 pills/6 g	6 g/t; b.i.d./t.i.d.

Yinxingye (YXY) tablet	Chinese pharmacopoeia (2010 edition)	Ginkgo Folium	12 mg/tablet; 24 mg/tablet;	24 mg/t; t.i.d.

Jinkui Shenqi (JKSQ) pill	State Drug Approval Document No: Z11020054	Aconiti Lateralis Rddix Praeparata (Fu Zi), Cinnamomi Ramulus (Gui Zhi), Achyranthis Bidentatae Radix (Niu Xi), Rehmanniae Radix (Di Huang), Corni Fructus (Shan Zhu Yu), Dioscoreae Rhizoma, Poria (Fu Ling), Aliamria Rhizoma (Ze Xie), Plantaginis Semen (Che Qian Zi), and Moutan Cortex (Mu Dan Pi)	6 mg/granule	b.i.d.

**Table 4:** Clinical indications of the CPMs achieving consensus.

CPMs	TCM symptom-based indications	Percentage of consensus (%)
TMGT granule	Dizziness	82.35
	Distending headache	73.53

QJDH capsule	Dizziness	97.06*
	Dazzling	94.12*
	Tinnitus	94.12*
	Soreness and weakness of waist and knee	88.23
	Insomnia	76.47
	Vexing heat in the chest, palms, and soles	76.47

BXTM pill	Heavy head	91.18
	Chest and epigastric fullness and distress	88.23

JKSQ pill	Nocturia	88.23
	Fatigue	79.41
	Waist soreness	79.41
	Dizziness	73.53
	Knee weakness	73.53

*P < 0.05.

## References

[1] Singh RB, Suh IL, Singh VP (2000). Hypertension and stroke in Asia: prevalence, control and strategies in developing countries for prevention. *Journal of Human Hypertension*.

[2] World Health Organization (2012). *World Health Statistics*.

[3] Yusuf S, Reddy S, Ôunpuu S, Anand S (2001). Global burden of cardiovascular diseases. Part I: general considerations, the epidemiologic transition, risk factors, and impact of urbanization. *Circulation*.

[4] Hu SS, Kong LZ, Gao RL (2012). Outline of the report on cardiovascular disease in China, 2010. *Biomedical and Environmental Science*.

[5] Khor GL (2001). Cardiovascular epidemiology in the Asia-Pacific region. *Asia Pacific Journal of Clinical Nutrition*.

[6] Vorster HH (2002). The emergence of cardiovascular disease during urbanisation of Africans. *Public Health Nutrition*.

[7] Gu DF, Reynolds K, Wu X (2002). Prevalence, awareness, treatment, and control of hypertension in China. *Hypertension*.

[8] Whitworth JA (2003). 2003 World Health Organization (WHO)/International Society of Hypertension (ISH) statement on management of hypertension. *Journal of Hypertension*.

[9] Cao HX, Wang YY (2011). Evidence-based guidelines of clinical practice in Chinese medicine internal medicine (internal medicine). *China Press of Traditional Chinese Medicine*.

[10] Dai L, Wang YJ (2006). Clinical study on the effect of niuhuang jiangya pill in treating hypertension with anxiety. *China Journal of Chinese Materia Medica*.

[11] Tan YS, Deng SM (2004). Study on clinical effectiveness and mechanism of compound qishao jiangya tablet in treating hypertension. *Chinese Traditional Patent Medicine*.

[12] Liu SX, Sun M, Luo YF (2004). Clinical study on the effect of niuhuang Jiangya capsule in treating primary hypertension. *Chinese Journal of Integrative Medicine*.

[13] Tang ZZ, Wang ZT (2004). Effect of radix salviae injection on hemorheology and blood lipid in primary hypertension. *Journal of Third Military Medical University*.

[14] (2010). *Chinese Pharmacopoeia Commission. Chinese Pharmacopoeia*.

[15] Green J, Thorogood N (2009). *Qualitative Methods for Health Research*.

[16] Rowe G, Wright G (1999). The Delphi technique as a forecasting tool: issues and analysis. *International Journal of Forecasting*.

[17] Gupta UG, Clarke RE (1996). Theory and applications of the Delphi technique: a bibliography (1975–1994). *Technological Forecasting and Social Change*.

[18] Lin JZ, Liu YY (2010). Clinical study on the effect of tianma gouteng yin in treating grade 1 hypertension with young and middle aged people. *Journal of Cardiovascular Disease*.

[19] Xiao JF, Cai JZ (2002). Clinical study on the effect of integrative medicine in improving life quality of aged patients with grade 2 hypertension. *Fujian Journal of Traditional Chinese Medicine*.

[20] Chen HB, Shan TH (2003). Clinical study on the effect of integrative medicine in treating aged patients with grade 3 hypertension of 72cases. *Henan Traditional Chinese Medicine*.

[21] Zhang TS, Han L, Wang L (2005). Relation of syndrome types to hypertension grades and risk stratification in patients with primary hypertension. *Chinese Journal of Clinical Rehabilitation*.

[22] Bai CJ, Zhou Y, Wang L, Zhang DL, Yang Y (2005). Delamination of cardiovascular risk factor, staging and grading of hypertension and the changing characteristics of blood lipids and hemorheological indexes in hypertensive patients with different syndromes of traditional Chinese medicine. *Chinese Journal of Clinical Rehabilitation*.

[23] Xie L (2010). Description of the incompatibility between Chinese patent medicines commonly used in clinical practice. *Beijing Journal of Traditional Chinese Medicine*.

[24] Chen KJ (2010). Study on the treatment of hypertension with integrated traditional and western medicine. *Chinese Journal of Integrative Medicine*.

[25] Fang XM, Li F (2008). Clinical study on the effect of tianma gouteng yin combining with captopril in improving life quality of hypertensive patients. *Chinese Journal of Integrative Medicine on Cardiovascular Disease*.

[26] Deng XG (2000). The thinking and methods on clinical diagnosis and treatment of hypertension with integrated traditional and western medicine. *Journal of Traditional Chinese Medicine*.

[27] Ebrahim S, Davey Smith G (1998). Lowering blood pressure: a systematic review of sustained effects of non-pharmacological interventions. *Journal of Public Health Medicine*.

[28] Stevens VJ, Obarzanek E, Cook NR (2001). Long-term weight loss and changes in blood pressure: results of the trials of hypertension prevention, phase II. *Annals of Internal Medicine*.

[29] Zhang DZ, Wang YF (2002). Clinical study on the effect of tianma gouteng granule in treating hypertension with 318 cases. *Heilongjiang Medical Journal*.

[30] Jia QM, Liu SY (2004). Clinical study on the effect of tianma gouteng granule combining with levamlodipine besylate tablets in treating hypertension with 34 cases. *Chinese Community Doctors*.

[31] Wang DQ, Wang W, Sun XF, Zhao DZ, Du GY (2005). Effect of Tianma Gouteng recipe on interfering LV and aortic hypertrophy in renovascular hypertension rats. *China Journal of Chinese Materia Medica*.

[32] Zhang ZQ (1999). Clinical study on the effect of qiju dihuang capsule in treating hypertension with yin deficiency and yang excess pattern. *New Journal of Traditional Chinese Medicine*.

[33] Ouyang YP (2002). Treatment and observation of 34 cases of elderly hypertension with verapamjl and qiju dihuang pills. *Hunan Guiding Journal of Traditional Chinese Medicine*.

[34] Liu SX, Sun M (2004). Clinical study on the effect of niuhuang jiangya capsule in treating primary hypertension. *Chinese Journal of Integrative Medicine*.

[35] Yu SY (2007). Clinical study on the effect of niuhuang jiangya pill in lowering blood pressure and influencing sympathetic activity. *China Journal of Chinese Materia Medica*.

[36] Liu SX, Sun M, Luo YF (2004). Clinical study on treatment of primary hypertension by niuhuang jiangya pill. *Chinese Journal of Integrative Medicine*.

[37] Dong S, Zhu Z, Zhang Y, Xu Z, Cheng J (1999). Determination of 11 metal elements in niuhuang jiangya wan by atomic absorption spectrophotometer. *Spectroscopy and Spectral Analysis*.

[38] Zhou JC, Li YP (2007). Effect of ginkgo leaf tablet on quality of life of hypertensive patients. *Journal of China Pharmacy*.

[39] Li CG, Zhang RH (2007). Clinical study on the effect of ginkgo leaf tablet combining with felodipine in treating hypertension with 96-case. *Clinical Medicine of China*.

[40] Liu QS (2008). Study on influence of ginkgo leaf tablet in ventricular remodeling of hypertension basing on regulating transforming growth factor-*β*1. *Chinese Journal of Cardiovascular Rehabilitation*.

[41] Li W (2005). Ginkgo leaf tablet caused one case of acute urticarial. *Chinese Journal of Misdiagnostics*.

[42] Li BT, Zhang JY, Zhang XM (2003). Clinical study on the effect of jinkui shenqi pill in treating hypertension with 68 cases. *Journal of Traditional Chinese Medicine*.

[43] Liu YL (2008). Clinical study on the effect of jinkui shenqi pill for influencing microalbuminuria in hypertension patient. *New Journal of Traditional Chinese Medicine*.

[44] Li Y (2004). Problems in the application of jinkui shenqi pill in clinical practice. *Lishizhen Medicine and Materia Medica Research*.

